# Therapeutic Management and Long-Term Outcome of Hyperthyroidism in Patients with Antithyroid-Induced Agranulocytosis: A Retrospective, Multicenter Study

**DOI:** 10.3390/jcm12206556

**Published:** 2023-10-16

**Authors:** Carlos García Gómez, Elena Navarro, Victoria Alcázar, Antonio López-Guzmán, Francisco Arrieta, Emma Anda, Betina Biagetti, Fernando Guerrero-Pérez, Carles Villabona, Andrés Ruiz de Assín Valverde, Cristina Lamas, Beatriz Lecumberri, José Antonio Rosado Sierra, Julia Sastre, Juan José Díez, Pedro Iglesias

**Affiliations:** 1 Department of Endocrinology and Nutrition, Hospital Universitario Puerta de Hierro Majadahonda, Instituto de Investigación Sanitaria Puerta de Hierro Segovia de Arana (IDIPHISA), 28222 Madrid, Spain; juanjose.diez@salud.madrid.org (J.J.D.); piglo65@gmail.com (P.I.); 2 Department of Medicine, Universidad Autónoma de Madrid, 28046 Madrid, Spain; 3 Department of Endocrinology and Nutrition, Hospital Universitario Virgen del Rocío, 41013 Sevilla, Spain; elena.navarro.sspa@juntadeandalucia.es; 4 Department of Endocrinology and Nutrition, Hospital Universitario Severo Ochoa, 28914 Madrid, Spain; victoria.alcazar@gmail.com; 5 Department of Endocrinology and Nutrition, Complejo Asistencial de Ávila, 05004 Ávila, Spain; alopezg@saludcastillayleon.es; 6 Department of Endocrinology and Nutrition, Hospital Universitario Ramón y Cajal, 28034 Madrid, Spain; arri68@hotmail.com; 7 Department of Endocrinology and Nutrition, Complejo Hospitalario de Navarra, 31008 Pamplona, Spain; emma.anda.apinaniz@navarra.es; 8 Department of Endocrinology and Nutrition, Hospital Universitari Vall d’Hebrón, 08035 Barcelona, Spain; bbiagetti@vhebron.net; 9 Department of Endocrinology and Nutrition, Hospital Universitari de Bellvitge, L’Hospitalet de Llobregat, 08907 Barcelona, Spain; ferguepe@hotmail.com (F.G.-P.); 13861cva@comb.cat (C.V.); 10 Department of Endocrinology and Nutrition, Complejo Hospitalario Universitario de Albacete, 02008 Albacete, Spainclamaso@sescam.jccm.es (C.L.); 11 Department of Endocrinology and Nutrition, Hospital Universitario La Paz, 28046 Madrid, Spain; beatriz.lecumberri@salud.madrid.org; 12 Department of Endocrinology and Nutrition, Hospital Universitario Getafe, 28905 Madrid, Spain; joseantonio.rosado@salud.madrid.org; 13 Department of Endocrinology and Nutrition, Complejo Hospitalario Universitario de Toledo, 45007 Toledo, Spain; jsastrem@sescam.jccm.es

**Keywords:** antithyroid drug-induced agranulocytosis (AIA), carbimazole (CBZ), methimazole (MMI), propylthiouracil (PTU)

## Abstract

Background: Antithyroid drug-induced agranulocytosis (AIA) (neutrophils <500/µL) is a rare but serious complication in the treatment of hyperthyroidism. Methodology: Adult patients with AIA who were followed up at 12 hospitals in Spain were retrospectively studied. A total of 29 patients were studied. The etiology of hyperthyroidism was distributed as follows: Graves’ disease (*n* = 21), amiodarone-induced thyrotoxicosis (*n* = 7), and hyperfunctioning multinodular goiter (*n* = 1). Twenty-one patients were treated with methimazole, as well as six patients with carbimazole and two patients with propylthiouracil. Results: The median (IQR) time to development of agranulocytosis was 6.0 (4.0–11.5) weeks. The most common presenting sign was fever accompanied by odynophagia. All of the patients required admission, reverse isolation, and broad-spectrum antibiotics; moreover, G-CSF was administered to 26 patients (89.7%). Twenty-one patients received definitive treatment, thirteen patients received surgery, nine patients received radioiodine, and one of the patients required both treatments. Spontaneous normalization of thyroid hormone values occurred in six patients (four patients with amiodarone-induced thyrotoxicosis and two patients with Graves’ disease), and two patients died of septic shock secondary to AIA. Conclusions: AIA is a potentially lethal complication that usually appears around 6 weeks after the initiation of antithyroid therapy. Multiple drugs are required to control hyperthyroidism before definitive treatment; additionally, in a significant percentage of patients (mainly in those treated with amiodarone), hyperthyroidism resolved spontaneously.

## 1. Introduction

Agranulocytosis (neutrophils < 500/µL) is a serious but rare complication associated with the use of antithyroid drugs in the treatment of hyperthyroidism. Antithyroid drug-induced agranulocytosis (AIA) is the most common cause of pharmacological agranulocytosis [1] and occurs in 0.1–0.5% of patients with hyperthyroidism who are treated for Graves’ disease [2,3]. The onset of AIA is usually early and generally occurs within the first 3 months of treatment initiation [4,5].

AIA is associated with different antithyroid drugs, such as carbimazole (CBZ), methimazole (MMI), and propylthiouracil (PTU), without clear differences between them [6]. Among the risk factors for developing AIA, the use of high drug doses, a genetic predisposition (HLA-B*38:02 and HLA-DRB1*08:03), and treatment with amiodarone have been previously reported [7,8,9]. Conversely, adverse prognostic factors for agranulocytosis include age (>65 years), severe neutropenia (neutrophils < 100/µL), intercurrent infection, and pre-existing comorbidities [10]. The use of granulocyte colony-stimulating factor (G-CSF) has been shown to be clearly beneficial in the management of AIA, significantly shortening recovery and hospitalization time [11].

The development of an AIA implies the need for definitive treatment of hyperthyroidism via surgery and/or radioiodine since continuation with antithyroid drugs should be avoided due to the risk of a new episode of AIA [4,7].

The type of definitive treatment of hyperthyroidism in patients with AIA has not been evaluated in detail in the previous literature. For this reason, in the present study, we set out to analyze the treatment choices and long-term results of hyperthyroidism in patients who have developed AIA based on a retrospective and multicenter analysis of cases treated in our country.

## 2. Materials and Methods

### 2.1. Patients

The particular design of this study was configured as a thorough retrospective analysis of the medical records corresponding to those patients who have experienced AIA in 12 hospital centers in Spain. In this study, a careful examination of the medical records was carried out with the aim of collecting and analyzing relevant data that would shed light on the relationship between the use of these drugs and the appearance of this serious complication. In order to enlist individuals with AIA, we employed diagnostic and procedural coding systems with the 9th and 10th revisions of the International Classification of Diseases-Clinical Modification, based on World Health Organization (WHO) criteria, ICD-9-CM and ICD-10-CM (diagnostic codes: E05 Thyrotoxicosis [hyperthyroidism]; D70.2 Drug-induced agranulocytosis; and T38.2 Antithyroid poisoning, adverse effect and underdosage) and by directly contacting the pharmacy services of each hospital, which keep records of adverse drug effects. For this purpose, the collaboration of the different Endocrinology and Nutrition and Admission and Clinical Documentation services of the different hospitals in our country was requested. Patients who developed agranulocytosis (neutrophils < 500/µL) under treatment with antithyroid drugs between 1994 and 2022 who met the following inclusion criteria were included in the study: age ≥ 18 years, hyperthyroidism under treatment with antithyroid drugs, treatment with antithyroid drugs during the diagnosis of neutropenia, and having a follow-up time equal to or greater than 6 months after the definitive treatment of hyperthyroidism (surgery or radioiodine) or after spontaneous normalization of hyperthyroidism.

### 2.2. Study Design

We used clinical and analytical data obtained during the patient visits to the medical offices of the different medical and surgical specialties (both at the times of clinical diagnosis of AIA and at medical check-ups after thyroid surgery or after radioiodine treatment). Informed consent of the participants was not required, as this was a retrospective analysis of data from routine clinical practice. The study was approved by the Drug Research Ethics Committee (CEIm) of the Puerta de Hierro Majadahonda Hospital (identification code: PIL-CAR-2020-01).

### 2.3. Statistical Analysis

Quantitative data were expressed as the median and interquartile range (IQR). Qualitative data were expressed as numbers (*n*) and percentages (%). The normality of each variable was assessed using the Kolmogorov–Smirnov test. For comparison of means between two groups, the Mann–Whitney U test was used (given *n* < 30). For the comparison of proportions, the chi-square test was used, and the Fisher’s exact test was used when there were fewer than 5 events per cell. We deemed a *p*-value < 0.05 to indicate statistical significance.

## 3. Results

### 3.1. General and Demographic Characteristics

A total of 29 patients were studied (23 women, 79.3%; 56.3 ± 15 years). Only six patients were older than 65 years. The clinical characteristics of the studied population are shown in Table 1.

No statistically significant differences in the clinical characteristics were observed between men and women. Coexistence with other autoimmune diseases was observed in seven patients (one case of vitiligo in the male group, as well as one case of Still’s disease, one case of IgA deficiency, one case of erythema nodosum, one case of autoimmune gastritis, one case of IgG Kappa monoclonal gammopathy of uncertain significance, and another case of unspecified autoimmune pathology in the female group).

### 3.2. Data Related to Hyperthyroidism

All of the patients started drug treatment immediately after the diagnosis of hyperthyroidism: 21 patients with MMI, 6 patients with CBZ, and 2 patients with PTU, with the median cumulative doses at the diagnosis of agranulocytosis being 1000 [717.5–1345.0] mg, 625 [550–1125.0] mg and 4775 mg, respectively. Antithyroid treatment was not resumed in any of the patients after the occurrence of agranulocytosis. After AIA was established, the group of patients was distributed as follows: 14 patients had subclinical hyperthyroidism, 10 patients had clinical hyperthyroidism, three patients had subclinical hypothyroidism, one patient had normal thyroid function, and one patient had clinical hypothyroidism.

Regarding other medical treatments that were administered for the management of hyperthyroidism after AIA, twelve patients were treated with corticosteroids (eight with prednisone and four with dexamethasone), nine patients were treated with Lugol solution, three patients were treated with cholestyramine, and one patient was treated with iopanoic acid. Two patients underwent plasmapheresis (one patient with Graves’ disease and one with hyperthyroidism secondary to amiodarone) to provide euthyroidism due to severe thyrotoxicosis and AIA. Central venous catheters were placed in all of the patients. Total blood volumes (TBVs) of the patients were calculated according to the Glicher rule (TBV [mL] = patient’s weight [kg] × blood volume estimate/kg [mL/kg]). Total plasma volume (TPV) was calculated using TBV and hematocrit (Hct) data (TPV [mL] = [1-Hct × TBV). Fresh frozen plasma (FPP) was used for the plasma exchange. The vital signs of the patients were checked before, during, and after plasmapheresis. One of the patients who underwent this treatment (the patient with Graves’ disease) required two sessions to obtain a significant decrease in circulating thyroid hormones. This patient ultimately required surgery for definitive treatment, whereas the patient with amiodarone-related hyperthyroidism resolved on his own.

For symptomatic control, 19 patients (70.4%) received treatment with beta-blockers (propranolol) at a mean dose of 40 [20–60] mg/day for a median of 40 [20.5–75] days. Twenty-one patients received definitive treatment; specifically, thirteen patients underwent surgery, nine patients received radioiodine, and one of the patients required both treatments. Of the total number of patients who had received definitive treatment with radioiodine, six patients developed hypothyroidism.

As for the remaining eight patients, in whom definitive treatment was not performed, six patients (four with amiodarone-induced thyrotoxicosis and two with Graves’ disease) had spontaneous normalization of thyroid hormone values, and the remaining two patients died of septic shock secondary to AIA. It should be noted that both of the deceased patients were older than 65 years, had multiple comorbidities (one of the patients had ischemic heart disease and the other patient had chronic kidney disease), and both had clinical hyperthyroidism at the time of AIA diagnosis.

### 3.3. Data Related to Agranulocytosis

The median time to development of the disorder was 6.0 (4.0–11.5) weeks. During the course of AIA, the most frequently presenting sign was fever, which was present in 26 patients (89.6%), accompanied on numerous occasions by odynophagia, which was present in 20 patients (70.0%). The clinical presentation did not differ between patients over and under 65 years of age. Prior to the initiation of thionamide treatment, all of the patients had a normal blood count. Bicytopenia with involvement of the erythrocyte and leukocyte series was observed in two patients; no cases of pancytopenia were observed in this study.

In cases of fever (>38 °C) or clinical–analytical data of infection, broad-spectrum antibiotic treatment (generally with beta-lactams) was started for approximately 8 ± 3 days while awaiting the microbiological results of the blood cultures. In 26 patients, G-CSF was concomitantly administered, especially in those patients who were considered at higher risk (older than 65 years, symptomatic, and pluripathological), and the commonly prescribed dosage was 300 μg/day administered subcutaneously. The mean time to use of G-CSF was 6.3 ± 3.6 days, whereas the mean time to recover leukocyte counts (>1500/µL) was 6.5 ± 3.7 days. Blood cultures were positive in four patients (Saccharomyces cerevisiae, Aspergillus, Staphylococcus Aureus, and Staphylococcus hominis). The average length of hospital stay was 8.9 ± 4.5 days.

## 4. Discussion

Hyperthyroidism is a relatively common disease, with an estimated prevalence between 0.2 and 9.3% in Europe [12,13]. A recent study in Navarra revealed a prevalence of hyperthyroidism in this area of 4.3% (5.6% in women; 3.0% in men) [12]. Its incidence in Europe is 51 cases per 100,000 inhabitants/year, with a ratio of approximately 5:1 female to male [13]. Graves’ disease (GD) is the most common cause of hyperthyroidism and occurs at all ages, but especially in women of reproductive age [12]. Graves’ hyperthyroidism is caused by autoantibodies to the thyroid-stimulating hormone receptor (TSHR) that act as agonists and induce excessive thyroid hormone secretion, thus releasing the thyroid gland from pituitary control [5,12]. Two clinical presentations of hyperthyroidism secondary to amiodarone have been distinguished [5,14]. Type 1, which is known as iodine-induced thyrotoxicosis, arises in thyroids with preexisting conditions (such as diffuse or nodular goiter and Graves’ disease) and is attributed to an augmentation in the synthesis and release of thyroid hormones. Conversely, type 2, which is referred to as pharmacologically induced destructive thyroiditis, manifests as a result of amiodarone’s cytotoxic impact on follicular cells, thus leading to the release of stored thyroid hormones in the colloid. In patients with type 1 amiodarone-induced hyperthyroidism, the therapeutic objective is to inhibit iodine organification and thyroid hormone synthesis. Typically, these patients exhibit a gradual response to thionamide therapy, due to the intrathyroidal iodine burden resulting from amiodarone metabolism, which may take from 3 to 5 months to occur [3,5]. It is worth noting that this classification of type 1 and type 2 disorders is not always definitive, as many cases involve mixed forms, wherein characteristics of both types coexist. Therefore, the initiation of thionamides and its subsequent discontinuation are contingent upon the clinical responses of the patients.

Thionamides have been the first-line treatment for hyperthyroidism for more than 50 years and, despite their efficacy, they are associated with a rare (but potentially) lethal effect known as agranulocytosis [1,2,3,4,5,6,7]. MMI and CBZ have a half-life of 4–6 h and a good intrathyroidal concentration (thyroid/plasma gradient of 1/100), so they can be prescribed in a single daily dose, which can improve therapeutic adherence [1,2,3]. PTU has a half-life of 1.5 h; however, it has the advantage that, at high doses, it inhibits the conversion of T4 to T3 in peripheral tissues by type 1 deiodinase, which increases its efficacy. This represents a very useful circumstance in cases of severe thyrotoxicosis, in which a more rapid improvement is desired [3,6]. The estimated prevalence of AIA is 0.1% to 0.5% according to previous studies [14,15]. Before starting thionamide treatment, there may be mild neutropenia and hypertransaminasemia, which typically resolve with the improvement of hyperthyroidism [16]. A total neutrophil count < 500/μL or a transaminase concentration at least five times the upper limit are relative contraindications for initiating antithyroid treatment [16]. None of the patients included in the current study presented with these two alterations before treatment.

Two mechanisms have been postulated to explain the pathophysiology of AIA. The first one would consist of the potential of some drugs to be oxidized and transformed into reactive species by neutrophils, inducing an immune response that culminates in their destruction. These reactions are mediated by myeloperoxidase, which is present in early granulocyte development [17]. The second mechanism, which reflects an immune nature and could also play a role in AIA, consists of the presence of circulating antibodies against differentiated granulocytes (ANCA) that would react against specific granules within granulocytes and could be one of the main mechanisms in PTU-induced agranulocytosis [18]. Multiple studies have attempted to clarify the existence of a genetic background in AIA. Chen et al. have shown that HLA-B*38:02 and HLA-DRB1*08:03 haplotypes constitute a risk factor for the development of AIA (odds ratio [OR]: 21.48 for HLA-B*38:02 carriers; OR: 6.13 for HLA-DRB1*08:03; and OR: 48.41 for those with both alleles) [9,19].

This individualization would constitute a progression towards precision medicine, in which patients carrying one of these haplotypes could opt for radioiodine or surgery as the first choice of therapy. However, due to the high costs associated with these advanced techniques, they have not yet become standard in routine clinical practice. None of the patients included in this study participated in a genetic study.

In our study, the median time to development was 6.0 (4.0–11.5) weeks, which is similar to that described in other literature [20]. In a series of 55 cases reported by Tajiri et al., none of the patients developed AIA after 70 days from the start of treatment [6], and only one case was observed after 13 months in the study by Tamai et al. [15].

Periodic monitoring of white blood cell counts in patients treated with antithyroid drugs is not recommended [6,20], although some authors have identified more than 75% of patients with granulocytopenia before the onset of symptoms after periodic monitoring of white blood cell counts [6].

Previous work has suggested the possibility that the adverse effects of MMI and CBZ are dose-dependent, whereas in the case of PTU, this evidence is even less robust [19,20]. In our cohort, no significant difference was observed between the different groups, given the homogeneity in the dose of treatments received. Most patients received MMI 15–20 mg/day; however, the initial dose of MMI was not collected in all of the patients so the frequency of AIA in relation to the starting dose cannot be determined. Until about the mid-1990s, the standard starting dose in the treatment of Graves’ disease was 30 mg MMI [21]; however, multiple subsequent studies have demonstrated the efficacy of lower doses of MMI (10–15 mg), which has been accompanied by a significant reduction in the frequency of adverse effects [21,22]. A meta-analysis by Nakamura et al. including 754 cases of AIA (726 cases related to MMI; 28 cases secondary to PTU) revealed that an MMI dose of 25.2 ± 12.8 mg increased the risk of AIA and was significantly associated with increased mortality [4].

The symptoms of AIA do not differ from those of agranulocytosis of other etiology. The most frequent presenting sign of AIA is fever [14], which was present in 26 patients in our sample (89.6%), with fever often accompanied by odynophagia. The results of Tajiri et al. showed that out of a total of 70 patients diagnosed with AIA, only 19 (27.1%) were symptomatic [6]. We are able to describe three patterns of AIA according to the literature: a first group of patients (classic agranulocytosis), in which data suggestive of infection leads us to the diagnosis; a second group (transitional agranulocytosis), in which the diagnosis is made by means of routine laboratory tests (wherein the patient is asymptomatic and will later develop infectious symptoms, despite the suspension of treatment with thionamides); and a last group of patients, in which no signs or symptoms are present during the whole process [14,23]. According to this theoretical classification, the majority of our cohort would fall into the first group described.

The recovery time of neutrophil numbers ranges from 7 to 24 days [11,22]. Regarding the use of G-CSF, there is controversy as to whether its use shortens the recovery time of leukocyte counts. The group of Andrès et al. reported leukocyte count recovery rates ranging from 6.8 days with the use of G-CSF to 11.6 days without the use of G-CSF [22]. In our sample, 26 patients (89.6%) received treatment with G-CSF, and the mean time to recovery of neutrophil counts (>1500/μL) was 6.5 ± 3.7 days, with no statistically significant differences with those patients who had not received G-CSF. Other groups such as Tajiri et al. [6] and Yang et al. [23] have described a reduction of approximately 4 days in the time to recovery of neutrophil counts with the use of G-CFS; however, in patients with severe agranulocytosis (<100/μL), these results were not observed [23].

Once agranulocytosis is established, there are several pharmacological alternatives to thionamides that can be used (both in monotherapy and in combination) to achieve hormonal control prior to definitive treatment. Given the high risk of cross-reaction between MMZ/CBZ and PTU (15–20%), it is not advisable to start another thionamide [4,7]. Beta-blockers attenuate the adrenergic symptoms associated with hyperthyroidism (palpitations, anxiety, heat intolerance, shortness of breath, and tremor) [24,25]. In a study by Toft et al., 100 patients with hyperthyroidism were treated with propranolol monotherapy prior to subtotal thyroidectomy without significant increases in morbidity and mortality [26]. Potassium iodide (Lugol) inhibits thyroid hormone synthesis (Wolff–Chaikoff effect) by decreasing thyroperoxidase activity, oxidation, and intra-thyroid organification, as well as reducing thyroid vascularization and the risk of bleeding during thyroidectomy [25]. Iodinated radiographic contrasts such as iopanoic acid (Colegraf^®^) and sodium ipodate (Orografin^®^) reduce the conversion of T4 to T3 by inhibiting the activity of type 1 deiodinase [24], but they are currently rarely used. Cholestyramine acts as a bile acid sequestrant that decreases the enteral reabsorption of thyroid hormones, thus decreasing their systemic circulation [25]. Glucocorticoids (dexamethasone) reduce the conversion of T4 to T3 by inhibiting type 1 deiodinase and stabilizing the follicular cell membrane [24,25]. Plasmapheresis represents an invasive procedure that exhibits certain risks and can induce blood clotting disorders due to the elimination of most of the coagulation factors that are present in plasma [27]. As a consequence of plasmapheresis, patients require a transfusion of fresh frozen plasma to restore the coagulation factors that have been lost while providing a new pool of thyroid hormone-binding proteins. It is important to note that therapeutic plasmapheresis, despite its apparent simplicity, can generate side effects that include hemolysis, anaphylactic shock, allergic reactions, infections, coagulopathies, bleeding, and a significant decrease in blood pressure [27]. It is critical to keep in mind that the impact of plasmapheresis in thyrotoxicosis is transient in nature, lasting from approximately 24 to 48 h. Therefore, it is imperative that definitive treatment be implemented once improvement in the patient’s clinical status has occurred. The guidelines of the American Society for Apheresis (ASFA) only postulate this treatment as a second therapeutic option in the case of thyroid storm and failure of medical treatment; however, they do not make specific recommendations for its use in other situations of hyperthyroidism [27].

The appropriate type of treatment to pursue for this disorder is currently unclear [2,4,17]. Although surgery controls hyperthyroidism at earlier time points [5], adequate control of hyperthyroidism before surgery is desirable through the use of combined pharmacological therapeutic regimens other than synthetic antithyroid drugs, and there is even the possibility of using plasmapheresis. When the patient is scheduled to undergo surgery, careful preoperative management is essential to optimize surgical outcomes [24]. Pre-treatment with thionamides in order to promptly restore euthyroidism prior to surgery is recommended to minimize potential complications, particularly for avoiding the risk of precipitating severe thyrotoxicosis (thyroid storm) during surgery [26]. The thyroid gland in patients with Graves’ disease is very vascular, and thyroidectomy can be associated with higher rates of bleeding compared to thyroids not affected with Graves’ disease [22,25].

On the other hand, radioiodine treatment needs more time to definitively control hyperthyroidism [5,17,23]. Radioiodine can exacerbate thyrotoxicosis [4,28]. Several studies have shown that thionamides administered after radioiodine (for example, in Graves’ disease) prevent the increase in thyroid stimulating antibodies 3-6 months post-treatment compared to no treatment [17,23]. Most patients reach euthyroidism, or in its absence hypothyroidism, 2–3 months after receiving a single dose of 12–15 mCi (445–555 MBq) [4,17]; however, up to 15% of patients may need a second dose of radioiodine [28]. Based on these premises, in our series, there was evidence of a preference for surgery as the primary treatment for overt hyperthyroidism. Most of the patients who underwent surgery had this disorder, whereas only one patient treated with radioiodine had severe hyperthyroidism.

We observed spontaneous remission of hyperthyroidism without the need for definitive treatment in six patients (four with amiodarone thyrotoxicosis and two with Graves’ disease). Notably, except in one case, none of these patients had severe hyperthyroidism at the time of AIA, thus suggesting that active surveillance could be a viable alternative in cases of mild hyperthyroidism secondary to amiodarone, in which the use of synthetic antithyroid drugs is contraindicated.

Mortality in these patients ranges from 4 to 6.2% [2,22], which is similar to the findings of our study (6.8%). Pearce et al. described a mortality rate of 18% [29]; however, Cooper et al. performed a systematic review of patients presenting with AIA between 1981 and 2003, and the estimated mortality in this study was approximately 2% [30]. A greater knowledge of the pathophysiology of this disorder, as well as the reduction of the dose of thionamides that was used and the optimization in the management of this entity, are probably the main causes of this important reduction in mortality.

Our study has the limitations inherent to retrospective analysis, such as the lack of some data on clinical and/or biochemical endocrine evaluations and a possible selection bias in favor of the most severe cases. As main strengths, it should be noted that there are few studies of AIA in the Caucasian population and that the collected sample comprises different etiologies of hyperthyroidism and explores its in-hospital management.

In conclusion, AIA is a serious complication associated with the use of antithyroid drugs in the treatment of hyperthyroidism that can currently be successfully managed in the hospital setting. Treatment consists of the withdrawal of antithyroid drugs, reverse isolation, broad-spectrum antibiotic coverage, use of G-CSF, and assessment of definitive treatment of hyperthyroidism. In our study, there was a greater indication for surgery as a therapeutic option for cases of severe hyperthyroidism after the AIA. In a significant proportion of patients treated with amiodarone, hyperthyroidism resolved without further intervention. Therefore, active surveillance in this subgroup of patients may be a viable option. An understanding of the clinical presentation and proper management of AIA could contribute to a reduction in its complications and mortality rate.

## Figures and Tables

**Table 1:** Clinical characteristics of the 29 patients diagnosed with antithyroid drug-induced agranulocytosis (AIA) distributed according to sex.

	Total	Males	Females
*n* (%)	29 (100%)	6 (20.6%)	23 (79.3%)
Age at diagnosis of AIA (years), mean ± SD	56.3 ± 15	65.0 ± 16	53.5 ± 14
Diabetes mellitus, *n* (%)	3 (10.3%)	0 (0%)	3 (13.0%)
Hypertension, *n* (%)	13 (44.8%)	3 (50%)	10 (43.5%)
Active smoker, *n* (%)	3 (10.3%)	0 (0%)	3 (13.0%)
Former smoker, *n* (%)	4 (13.7%)	3 (50%)	1 (4.3%)
Autoimmune diseases, *n* (%)	7 (24.1%)	1(16.7%)	6 (26.1%)
Graves’ disease, *n* (%)	21 (72.4%)	4 (66.7%)	17 (73.9%)
Amiodarone-induced hyperthyroidism, *n* (%)	7 (24.1%)	2 (33.3%)	5 (21.7%)
Hyperfunctioning multinodular goiter, *n* (%)	1(3.4%)	0 (0%)	1(4.3%)
Methimazole, *n* (%)	21(72.4%)	4 (66.7%)	17 (73.9%)
Carbimazole, *n* (%)	6 (20.7%)	1 (16.6%)	5 (21.7%)
Propylthiouracil, *n* (%)	2 (6.9%)	1 (16.6%)	1 (4.3%)

## Data Availability

The data presented in this study are available on request from the corresponding author.
